# 先进分子印迹技术及其前沿应用专辑引言

**DOI:** 10.3724/SP.J.1123.2025.12004

**Published:** 2026-01-08

**Authors:** Qiong JIA, Kaiguang YANG

分子印迹技术作为一种模拟天然抗原-抗体相互作用机制的仿生识别技术，通过在人工化学材料网络中构建与模板分子在空间结构及功能基团上精准匹配的空穴，实现对目标分子的高特异性识别。据此开发的分子印迹材料，具有制备简单、成本低廉以及稳定性好等优势，在环境监测、催化、食品安全、生物医学等领域展现出巨大的应用潜力。

近年来，分子印迹技术展现出更高效、更智能、更环保的发展趋势，一系列新策略和新材料应运而生。为总结该领域的最新研究成果，探讨技术前沿与未来研究方向，受《色谱》期刊委托，我们组织了先进分子印迹技术及其前沿应用专辑，邀请了国内高等院校及科研院所的相关领域专家学者为本刊撰稿。

本专辑经过同行专家严格评审，最终录用聚焦1 篇、视角1 篇、专论与综述9 篇、研究论文6 篇，分别安排在2026 年第1 期和第2 期出版。在材料制备与方法创新方面，涉及金属有机框架、石墨烯气凝胶、纳米酶等新材料以及电场辅助、绿色合成、细胞印迹等前沿技术；在应用研究方面，涉及环境污染物筛查、催化降解、疾病生物标志物精准识别、循环肿瘤细胞高效捕获等重要领域。我们衷心希望通过本专辑的出版，能够全面展现分子印迹技术的最新进展，为相关领域的研究人员提供有益的借鉴与启示，进一步推动分子印迹技术的深入发展与实际应用。

衷心感谢各位作者及审稿专家的鼎力支持和奉献！

本专辑客座主编

吉林大学 贾琼 教授

中国科学院大连化学物理研究所 杨开广 研究员

2025年11月29日

